# MiRNA and TF co-regulatory network analysis for the pathology and recurrence of myocardial infarction

**DOI:** 10.1038/srep09653

**Published:** 2015-04-13

**Authors:** Ying Lin, Vusumuzi Leroy Sibanda, Hong-Mei Zhang, Hui Hu, Hui Liu, An-Yuan Guo

**Affiliations:** 1 Hubei Bioinformatics & Molecular Imaging Key Laboratory, Department of Biomedical Engineering, Key Laboratory of Molecular Biophysics of the Ministry of Education, College of Life Science and Technology, Huazhong University of Science and Technology, Wuhan, 430074, China

## Abstract

Myocardial infarction (MI) is a leading cause of death in the world and many genes are involved in it. Transcription factor (TFs) and microRNAs (miRNAs) are key regulators of gene expression. We hypothesized that miRNAs and TFs might play combinatory regulatory roles in MI. After collecting MI candidate genes and miRNAs from various resources, we constructed a comprehensive MI-specific miRNA-TF co-regulatory network by integrating predicted and experimentally validated TF and miRNA targets. We found some hub nodes (e.g. miR-16 and miR-26) in this network are important regulators, and the network can be severed as a bridge to interpret the associations of previous results, which is shown by the case of miR-29 in this study. We also constructed a regulatory network for MI recurrence and found several important genes (e.g. DAB2, BMP6, miR-320 and miR-103), the abnormal expressions of which may be potential regulatory mechanisms and markers of MI recurrence. At last we proposed a cellular model to discuss major TF and miRNA regulators with signaling pathways in MI. This study provides more details on gene expression regulation and regulators involved in MI progression and recurrence. It also linked up and interpreted many previous results.

Myocardial infarction (MI) is a leading cause of death in the world and also known as heart attack, which often leads to ischemia and cardiac necrosis due to blockage of a coronary artery^1^. The prevalent profiles of MI are believed to be multifaceted and to be impelled by many genes, which individually have a small effect, and hence function alone or in collaboration with other genes or environmental factors^2^. Hundreds of studies reported that many genomic regions and genes are associated with or be risk loci of MI, such as the 9p21.3 genomic region^3^,^4^. The international Myocardial Infarction Genetics Consortium published a GWAS study for early-onset myocardial infarction and identified 9 significant loci, including regions near MRPS6-SLC5A3-KCNE2, PHACTR1, WDR12, CELSR2-PSRC1-SORT1, CXCL12, MIA3, LDLR and PCSK9^5^. Recurrence of MI after a first occurrence will significantly increase the risk of death in patients^6^,^7^. Strategies for prediction and prognosis of a recurrent MI will prolong survival in post-MI survivors^7^. It has been report that the Phospholipase A2 expression and plasma myeloperoxidase level is associated with recurrence of cardiac events after acute MI (AMI)^8^,^9^. Recently, a group reported that epithelial-to-mesenchymal transition pathway and cholesterol transport genes were associated with long-term recurrent events following first MI through studying the transcriptome of AMI patients with or without recurrent events^10^. However, the gene regulation especially their regulatory network for the development and recurrence of MI is unclear.

MicroRNAs (miRNAs) are small noncoding RNAs suppressing gene expression via imperfect base pairing to the 3′ untranslated region (3′UTR) of target mRNAs^11^. A number of reports have proved that miRNA dysregulations were associated with cardiac events by influencing myocardial contractile, myocytes apoptosis, myocardial fibrosis and angiogenesis, thus making them plausible diagnostic, prognostic and therapeutic markers^12^. Overexpression of miR-101a can mitigate interstitial fibrosis and the deterioration of cardiac performance by decreasing c-Fos and its downstream TGFB1^13^. Smad3 signal negatively modulated miR-29b expression, miR-29b as a regulator of cardiac fibrosis could efficiently inhibit fibrosis-related genes expression in cardiac fibroblasts^14^,^15^. Another study evaluated a critical role of NF-κB-mediated miR-30b modulation in Ang II-stimulated cardiomyocytes targeting Bcl-2^16^. Transcription factors (TFs) are paramount regulators of gene expression in living organisms^17^, and also play important roles in MI. Such as EGR1, ATF3, ATF4, MYC and FOS were considered as the key TFs related to the development of AMI^18^. TF and miRNA may mutually regulate each another hence forming feedback loops (FBLs), or regulate a shared target gene to form feed-forward loops (FFLs)^11^,^19^,^20^. Both miRNA-mediated FBLs and FFLs are significant and recurrent network motifs, which play important roles in gene regulation in mammalian genomes^21^,^22^. A FFL is a motif, in which regulator A regulates another regulators B, and both regulators A and B regulate a common target C^21^. This will enhance the robustness of gene regulation. Hence, the TF-miRNA regulatory network analysis will be helpful to decipher the role of major regulators and regulation in MI.

In this study, by curating MI related candidate genes and miRNAs from literatures, we constructed a comprehensive MI-specific miRNA-TF co-regulatory network. Based on our network analysis and literature survey, we found that our network can be a bridge and provide potential clues to interpret the associations of previous results from different groups. We also investigated the regulatory factors for differentially expressed genes between recurrent and non-recurrent AMI patients. This work will be helpful to reveal the occurrence and recurrence mechanism of MI, as well as provide potential markers.

## Results

### The MI miRNA-TF co-regulatory network and its characteristics

Aiming to explore the miRNA and TF co-regulatory network in MI, it is indispensable to determine the MI related genes, miRNAs and TFs. So we first manually collected and curated 83 experimentally verified MI related miRNAs and 153 MI related genes from databases and publications as described in Methods. All these miRNAs are conserved in vertebrate and mammal genomes; 45 miRNAs are in host genes and 38 are intergenic miRNAs (Supplementary Table S1). All these genes play critical roles with dysregulated expression in MI patients, cell lines and/or animal models (Methods and Supplementary Table S2).

**Figure 1:**
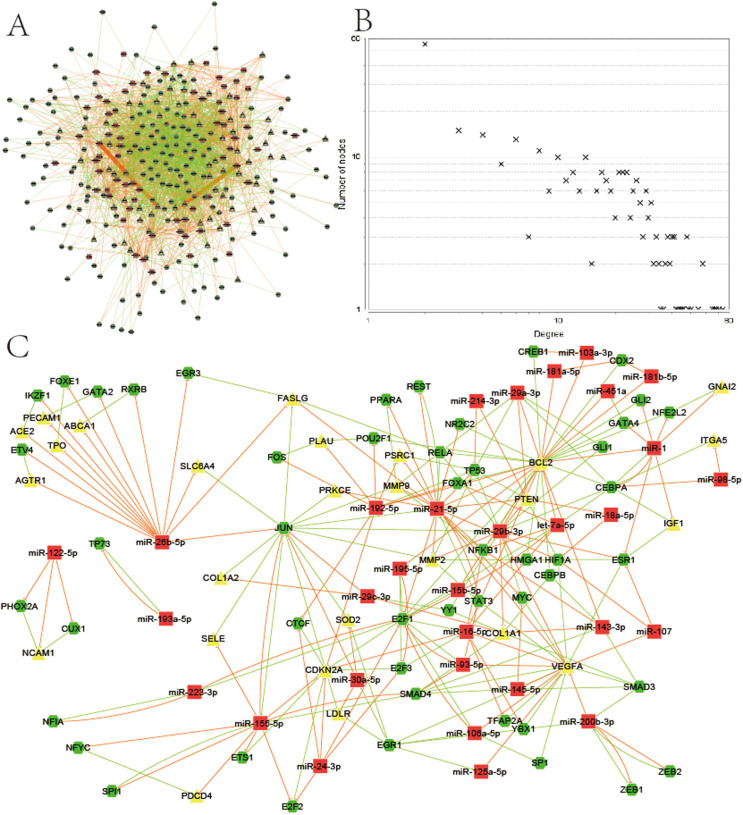
The MI miRNA-TF co-regulatory network and its characteristics. (A) Graphical representation of the MI miRNA-TF co-regulatory network. (B) Node degree distribution in the network. (C) Verified FFLs in the MI network. Red rectangles: MI related miRNAs. Yellow triangles: MI related genes. Green hexagon: TFs. The edge colors represent different relationships: red for the repression of miRNAs to genes or TFs, green for the regulation of TFs to genes or miRNAs.

We investigated the FFLs and feedback loops among these genes, and then constructed the miRNA-TF co-regulatory network in MI as described in the Methods section and Supplementary Fig. S1. The resultant network included 304 nodes and 2492 edges (Fig. 1A), which recruited 66 (43%) of the 153 MI related genes, 69 (83%) of the 83 MI related miRNAs and 169 human TFs. Given the high certainty of association between our candidate genes and the disease, we considered it as a representative regulatory network in MI. We studied the node degree-distribution to assess the overall characteristics of the network. In this regulatory network, the node distribution was significantly right-skewed (Fig. 1B); implying that only a few nodes had a significantly higher node degree in the network.

In Table 1, we summarized the FFLs in the MI network. The detailed miRNA and TF target regulation were in Supplementary Dataset1 and the FFLs information in Supplementary Dataset2. Among the 2,382 merged FFLs, 1424 (59.8%) are TF-FFLs, in which TF is the main regulator, 815 (34.2%) belonged to miRNA-FFLs, and 143 (6%) belonged to composite-FFLs. The 6% composite-FFLs comprised of 32 (48.5%) MI related miRNAs and 32 (18.9%) TFs regulating 51 (77.3%) MI related genes. This indicates that composite-FFLs drafted more than half of the MI related genes via a few regulators. Our observations suggest that a majority of the MI related genes is regulated in multiple ways. We also constructed the verified FFLs network in MI using the verified regulations as described in the Methods section. The verified FFLs network included 106 nodes and 244 edges, and it recruited 26 (39.4%) of the 66 MI related genes, 31 (44.9%) of the 69 MI related miRNAs and 49 TFs. Fig. 1C illustrates the verified network which constitutes only a small percentage of the overall predicted miRNA-TF co-regulatory network in MI; this observation testifies to the novelty of the miRNA-TF co-regulatory network and its potential for deciphering new regulatory mechanisms.

### Hubs and enriched pathways show the reliability of the network

**Figure 2:**
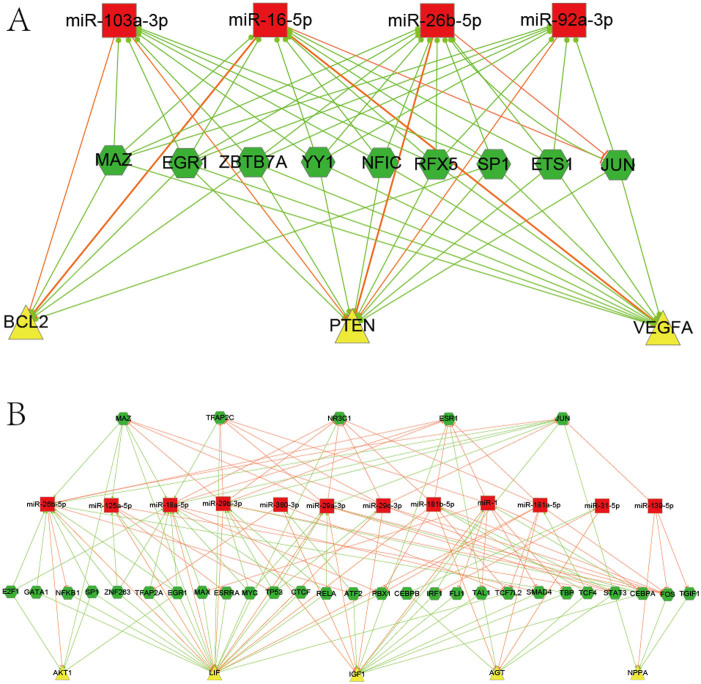
Subnetwork of representative hubs and pathway. (A) Subnetwork among hubs in the MI miRNA-TF co-regulatory network. (B) The NFAT and Hypertrophy of the heart specific regulatory network. The means of different nodes and edges are the same as Figure 1.

Hubs play critical roles in a network and hub analysis is an effective way to understand the network. According to the method, we identified 3 hub genes (PTEN, VEGFA and BCL2), 4 hub miRNAs (miR-103/16/92a/26b) and 9 hub TFs (EGR1, JUN, MAZ, NFIC, SP1, ZBTB7A, RFX5, ETS1 and YY1). Fig. 2A shows the subnetwork of these hub components, which are closely related to MI. The expression of VEGFA has been linked to the induction of angiogenesis, which is an essential process for cardiomyocyte recovery after MI event^23^. The antioxidant role of BCL2 is essential for the cardioprotection achieved by ischemic adaptation, while the overexpression of miR-16 was at high risk of impaired LV contractility^24^,^25^. Bridging these results with our network, we proposed miR-16 may promote the MI event by targeting the cardio-protective genes, BCL2 and VEGFA. The inhibition of PTEN enhances PI3K/Akt signaling and could prevent myocardium from ischemia-reperfusion injury^26^. Our results partially confirm that miR-26 is cardio-protective as it represses PTEN. Previous studies showed that expression levels of TFs like c-fos, c-jun, c-myc, Egr-1 were linked to the extent of remodeling and hypertrophy after MI^27^ and we believed that hub TFs of our network could play key roles in MI. So our network may provide some potential clues to interpret the associations of previous results from different groups.

Enrichment analysis is an effective way to understand functional genes and network modules. Firstly, we carried out the functional enrichment analysis of all TFs in the MI miRNA-TF co-regulatory network and identified six highly enriched biological processes (Table 2). These enriched pathways could give us more insights for the roles of TFs in MI. For example, the anti-apoptosis signaling pathway was linked to 73 nodes and 208 edges which consisted of 9 TFs (CEBPB, NKX2-5, ESR1/2, NFKB1, STAT5A, TCF7L2, MYC and RELA). It has been reported that significant numbers of myocytes die by apoptosis during MI and apoptosis plays a role in the process of tissue damage after MI^28^. We further examined the enriched pathways for the 66 MI related genes involved in the network and identified 6 significantly enriched pathways (P-value < 0.01) (Table 2). Among them, three were directly related to MI, which are NFAT and Hypertrophy of the heart, Focal adhesion and ECM-receptor interaction^29^,^30^,^31^. We selected the NFAT and Hypertrophy of the heart (Transcription in the broken heart) pathway for further analysis as it is a major pathway in pathological remodeling and hypertension^29^. Five genes (AKT1, LIF, IGF1, AGT and NPPA) in our network belong to this pathway and their subnetwork was shown in Fig. 2B. We defined it as the NFAT and Hypertrophy of the heart specific regulatory network, which included 123 edges, 5 genes, 12 miRNAs and 32 TFs. These 12 miRNAs might be involved in the NFAT and Hypertrophy of the heart pathway, providing a potential pool for further experimental determination of miRNAs.

### Subnetwork for miR-29 as an example to interpret the significance of the network

**Figure 3:**
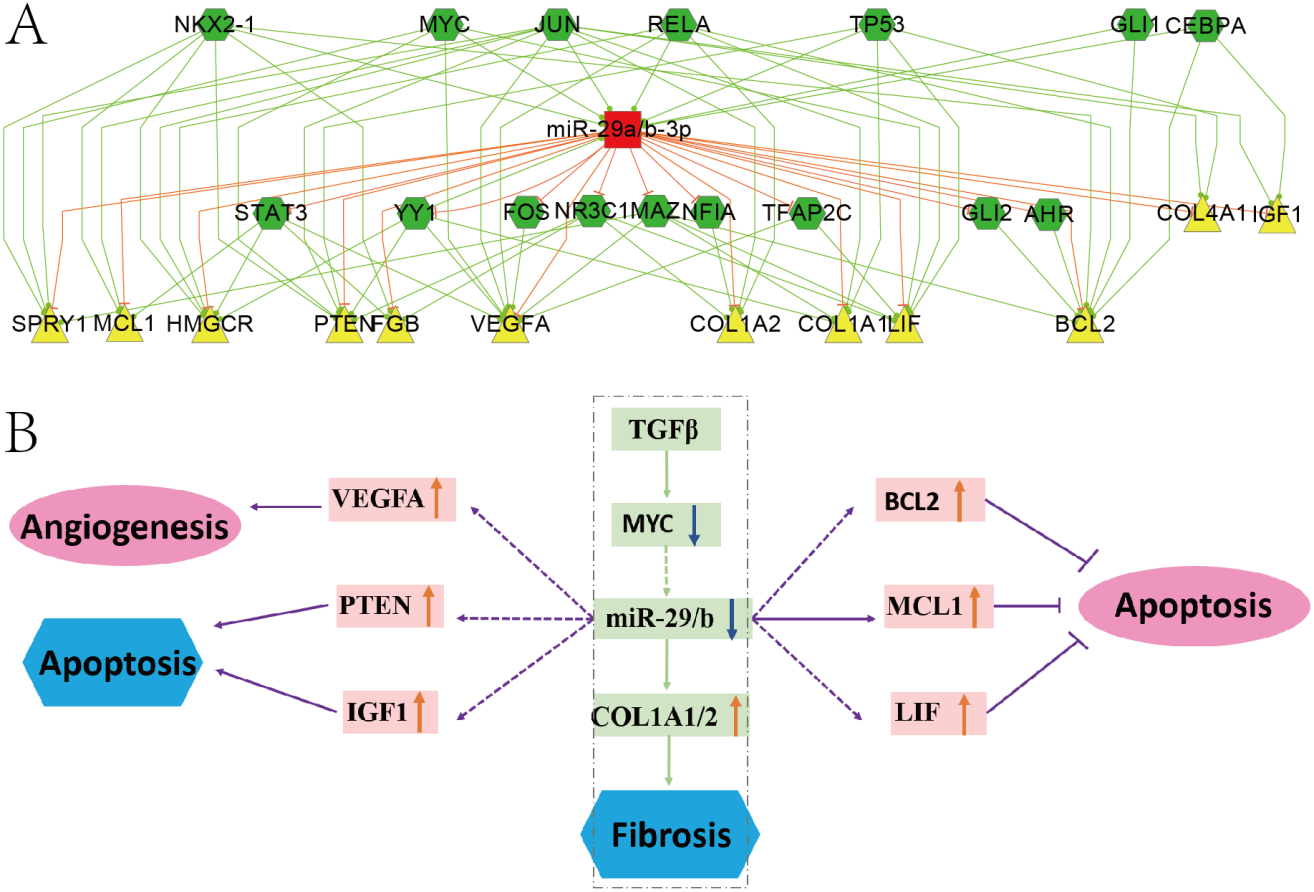
The miR-29a/b subnetwork and its model in MI. (A) MiR-29a/b subnetwork. The means of different nodes and edges are the same as Figure 1. (B) Model of miR-29a/b involved in the incidence of MI. Rectangular represents regulators. Pink Oval and blue hexagon represents positive and negative effects, respectively. The orange up arrow and blue down arrow indicate the expression status of corresponding regulators. Solid and dashed arrows are the verified and predicted regulatory relationships, respectively.

The MI miRNA-TF co-regulatory network were too complex to analyze, thus we chose the representative subnetwork of miR-29 to study the significance of the network. The miR-29 families emerged as promising contributors as they had 3 members in our network, and a large number of studies showed that members in miR-29 family were related to the pathogenic mechanism of MI^14^. Fig. 3A showed the FFL subnetwork of miR-29a/b combined TFs and MI genes. Combining this subnetwork and published results, we proposed a model that miR-29 involved in the incidence of MI (Fig. 3B). It further confirmed that our network can be a bridge to linkup and explain many results from previous studies.

Van Rooij et al. reported that miR-29 family members were down-regulated in fibroblasts by TGFβ^14^, and TGFβ also reduced the expression of MYC gene^32^. Our network predicted that MYC regulated the expression of miR-29, which may mediate the down-regulation of miR-29 by TGFβ. Cardiac fibrosis is a major aspect of the remodeling process after MI, many studies concluded that miR-29 acts as a regulator of cardiac fibrosis and might represent a therapeutic target for tissue fibrosis in general^14^,^33^. Our results confirmed the role of miR-29 in fibrosis through its targets COL1A1/COL1A2^34^ .We also found miR-29 could inhibit MCL1 in the subnetwork, which is consistent with reports that antagomirs against miR-29 increased Mcl-1 expression and significantly reduced myocardial infarction size^35^. In addition, our results also proposed several important MI genes were targets of miR-29, such as BCL2, PTEN, VEGFA and LIF, which are involved in the apoptosis/anti-apoptosis, angiogenesis, cardiac myocyte death and neovascularization^23^,^24^,^26^,^36^. These may imply more comprehensive roles of miR-29 involved in MI.

### The miRNA-TF co-regulatory network for MI recurrence

**Figure 4:**
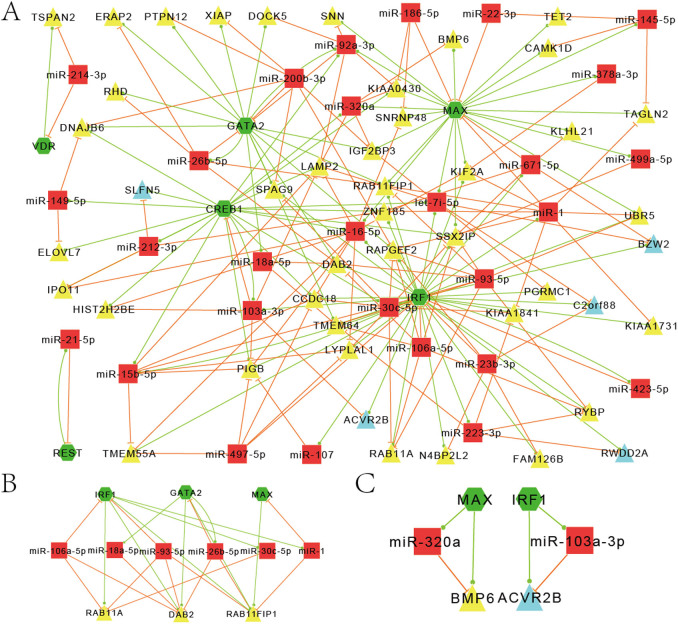
MiRNA-TF co-regulatory network associated with MI recurrence. (A) Graphical representation of the miRNA-TF co-regulatory network associated with MI recurrence. (B) Specific regulatory network of Endocytosis in MI recurrence. (C) FFLs for two genes in the recurrence of MI. Red rectangle represents miRNAs and green hexagon represents TFs. Yellow and blue triangles are genes down- and up-regulated in MI patients with recurrence, respectively.

Survivors of MI are at a high risk of recurrent infarctions and early prediction of the recurrence will be very important for patients. Based on the results of a group from Mayo Clinic^10^ and using a stricter cutoff, we obtained 100 differentially expressed genes and 31 differentially expressed TFs between AMI patients with and without an adverse recurrent at 18-month follow-up (Supplementary Table S3). These differentially expressed genes and TFs formed 117 FFLs (57% TF-FFLs, 33% miRNA-FFLs and 10% composite-FFLs) combined with MI related miRNAs. We tested the significance of these differential expressed genes combined MI miRNA and TF to form FFLs by random simulation test (see Method) and obtained a P-value 0.0002, indicating that the 100 differential expressed genes are significantly related to MI or MI recurrence. Then, we constructed a MI recurrence related network based on these FFLs aiming to predict some recurrent related regulatory modules. The resultant network included 79 nodes and 210 edges (45 genes, 28 miRNAs and 6 TFs) (Fig. 4A).

Among these 45 recruited genes, most of them are down regulated in patients with recurrence but only 5 genes (ACVR2B, BZW2, Clorf228, RWDD2A, and SLFN5) are up-regulated. Functional analyses of these genes revealed Endocytosis, Cytokine-cytokine receptor interaction and TGFβ signaling pathway as the most representative pathways. Previous studies show endocytic control of ion channel density as a target for cardiovascular disease^37^, and Endocytosis specific regulatory network was linked to 3 major TFs (IRF1, GATA2 and MAX), 6 miRNAs and 3 genes (DAB2, RAB11A and RAB11FIP1), which significantly down-regulated in patients with recurrence (Fig. 4B). We considered that the abnormal expression of those regulators and genes may be potential predictor of the risk of MI recurrence, and the original paper also confirmed that the abnormal expression of the DAB2 influence the severity of AMI through disruption of cholesterol transport^10^. TGFβ signaling is a crucial mediator in the pathogenesis of post-infarction remodeling^38^,^39^ and Cytokine-cytokine receptor interaction may be involved in the cardioprotective effects of curcumin^31^. It is notable that ACVR2B and BMP6 genes participate in both Cytokine-cytokine receptor interaction and TGFβ signaling pathway. Both MAX and BMP6 in the MAX/miR-320a/BMP6 FFL were decreased in patients with recurrence and miR-320 was also reported down-regulated in MI^34^. Thus, we proposed that the FFLs of ACVR2B and BMP6 may be potential biomarkers for the recurrence of MI (Fig. 4C). It will be very interesting to verify them by further experiments.

## Discussion

In this study, we investigated the co-regulation of miRNAs and TFs involved in the pathology as well as the recurrence of MI. We envisioned a computational framework to construct, investigate a regulatory network and thus identified some promising FFLs and regulators in MI.

Our work shows that it is possible and largely beneficial to process multiple types of data to construct a comprehensive and reliable co-regulatory network of MI. We concerned the regulation from all TFs to avoid losing some regulation but only use that from MI related miRNAs to reduce too much false regulation. We predicted the TF and miRNA mediated regulatory motifs in MI, including FBLs and FFLs, which are prevalent and significant motifs in gene regulation^11^,^22^. Cui et al. reported that genes with more TF-binding sites have a higher probability of being targeted by miRNAs and have more miRNA-binding sites on average^40^. We also found that many MI genes were predicted to be regulated by many TFs and miRNAs, such as LIF, IGF1 and AGT genes in Figure 2B. Many FFLs in our MI network are miRNA-FFLs, in which miRNA as the main controller regulates both TF and gene. This is consistent with previous report that miRNAs prefer to target downstream network components such as TFs^41^. The FFL analysis is likely a powerful tool to investigate regulatory mechanisms of MI progression at both the transcriptional and translational levels. The resultant network showed high specificity, for the hubs and the enriched pathways are functionally related with MI. Especially not only the hub genes and miRNAs (PTEN, VEGFA, miR-26/16) that are curated from publications but also the hub TFs (EGR1, JUN, SP1, YY1 etc.) all play important roles in MI^26^,^27^,^42^.

**Figure 5:**
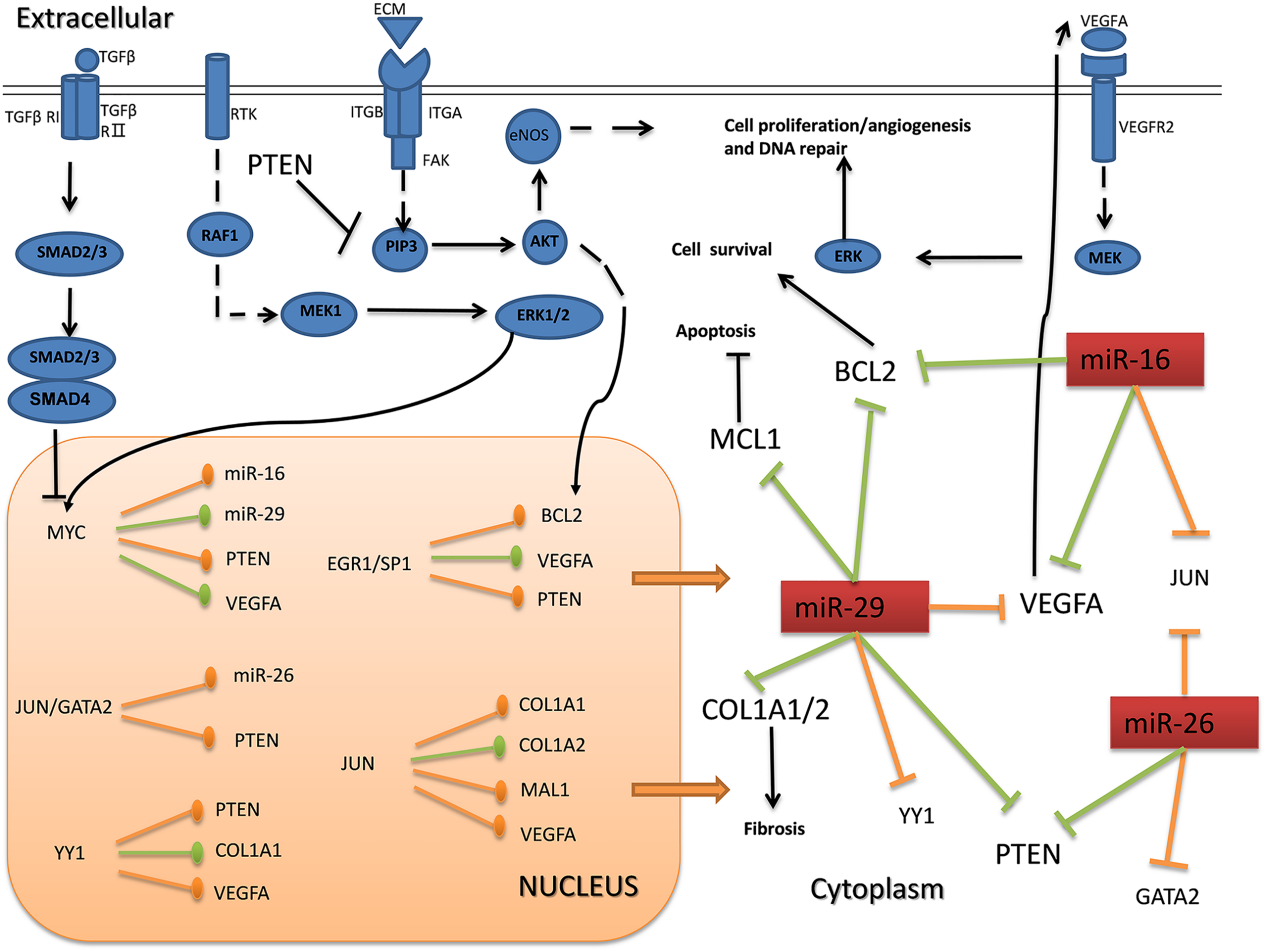
Cellular Model of essential components in MI progression. The graph illustrates the proposed model of MI progression co-regulated by miR-16-5p, miR-26b-5p, miR-29a-3p and the TFs; JUN, YY1, EGR1, SP1, GATA2 and MYC. These regulators control components of the VEGF signaling pathways, PI3K-Akt signaling pathways, MAPK signaling pathway and TGFβ signaling pathway. Red rectangle represents miRNAs. Regulations by arrows in orange and green are predicted and verified regulations, respectively.

Second our network is informative and could be used as a bridge to linkup many published results, which is helpful to bench researchers. For example the subnetwork of miR-29 linked several pathways and revealed its multifaceted effects in the pathology of MI. Thus we further try to interpret the possible function of the core sub-network consisted by key components of the network based on reviewing some related papers. We found that these promising regulators govern a plethora of cellular processes and were related to the pathology of MI via several representative pathways: VEGF signaling pathway^43^, MAPK signaling pathway^44^, TGFβ signaling pathway and PI3K-Akt signaling pathway^26^,^38^. The relations among them were shown in Fig. 5, and could be considered a possible model of MI, which may provide potential theoretical guidance for further research. Of course the model could not be the ultimate model for MI, and it is just the summary of the regulation among the key components in the verified network, which is a small part of the total network.

Furthermore, we analyzed the regulation relationship for the differentially expressed genes between recurrence and no recurrence AMI patients. First we found it is interesting that there are few overlapping genes between these genes and the curated MI genes. This might imply that different mechanisms are involved in the pathology and recurrence of MI. Indeed the enriched pathways are different. This is also the reason that we independently construct the co-regulatory network for MI recurrence. In this network we found some regulatory modules which would be used as potential markers. Taking the regulation of BMP6 by miR-320a as example, BMP6 has a significantly down expression in the MI patients with recurrence, it's consistent with the previous reported that over expression of miR-320 could increase cell death and apoptosis in cardiomyocyte^35^.

In summary, here we offered more details with regards to the molecular regulation involved in MI progression. Many regulations in our FFLs can be the junctions of published results by different groups. Some targets of the miRNAs in our network participated in multiple signal pathways, which may establish cross-talks among these pathways. We believe that our results identified some essential gene regulatory network modules, which will enhance our understanding of gene regulation mechanism in MI.

## Methods

### MI candidate genes and miRNAs

To collect genes involved in the pathology of MI, we compiled MI related genes from three sources. These sources included the Coronary Artery Disease gene database (CADgene, http://bioinfo.life.hust.edu.cn/CADgene2/)^45^, PUBMED search using keywords “(myocardial OR acute myocardial infarction) AND genes”, and results from MI genome wide association studies (GWAS)^3^,^4^,^46^,^47^,^48^. We mapped the genes to their Entrez gene symbols and eventually obtained 153 unique genes. Based on the study of Suresh et al.^10^, we obtained 559 differentially expressed genes between AMI patients with and without recurrent events at 18 months follow-up (p-value < 0.05). These genes include 31 TF genes. Making a stricter filter by changing the cutoff of p-value (p-value < 0.01), we obtained 100 MI recurrence related genes.

To collect a set of dysregulated miRNAs in MI, we conducted an extensive literature search for studies that directly assessed miRNA dysregulation in MI patients. We originally searched PUBMED using the keywords “(myocardial OR acute myocardial infarction) AND miRNAs”, then we also searched the miR2Disease^49^, and HMDD^50^ databases for relevant articles using the keywords “myocardial OR acute myocardial infarction”. We retrieved 83 miRNAs^51^,^52^,^53^, which were all mapped to unique mature miRNAs based on human miRNAs from miRBase.

### Identification of miRNA and TF targets

We merged the miRNA targets by both predicted and experimentally verified targets as described in our previous review paper^11^. They include predicted miRNA targets by utilizing the overlapped results from TargetScan (version6.2, June 2012) and miRanda (Release Date: September 2010)^54^,^55^ and experimentally verified miRNA targets from miR2Disease (Release Date: March 2011), miRTarBase (2013, version 4), miRecords (April 27, 2013) and TarBase (TarBase_V6, Jan 2012)^56^. In order to predict regulatory interactions between TF and gene/miRNA, we obtained predicted transcription factor binding sites (TFBS) data from the UCSC genome browser and expected the TFBSs to be preserved among vertebrates. To reduce the rate of false positive prediction, we set a Z score of 2.33 as a stringent cutoff for high value TFBSs. Additionally, we also incorporated TF targets from ChIP-Seq and ChIP-chip data, which were curated from the ENCODE project (http://genome.ucsc.edu/ENCODE/)^57^. Verified TF targets by experiments were extracted from TRANSFAC database (release 2013.4).

### The generation of network and subnetwork

After deciphering miRNA-gene/TF and TF-gene/miRNA regulatory relations, we utilized in-house scripts to construct the MI miRNA-TF co-regulatory network organized according to the miRNA-TF-gene FFLs and miRNA-TF FBLs. We constructed several major networks including MI-specific miRNA-TF network, verified MI network, hub subnetwork and recurrence related network. For the subnetwork of the NFAT and Hypertrophy of the heart (Transcription in the broken heart) signaling pathway, we firstly collected genes belonging to the pathway from BIOCARTA (www.biocarta.com), then merged those FFLs that included at least one gene involved in the pathway. These networks were visualized using Cytoscape (version 2.8)^58^.

### The hub component and pathway enrichment analysis

To determine network hubs, we sorted the nodes by descending according to their degrees, and then considered the top 5 percent of miRNAs, TFs and genes as the hub components. To identify pathways overrepresented in MI related genes from the MI composite regulatory network, we performed a pathway enrichment analysis using the Database for Annotation, Visualization and Integrated Discovery (DAVID) v6.7^59^. A p-value < 0.01 was adopted as the cutoff for enriched GO terms or pathways in Gene Ontology or KEGG.

### Statistical tests of FFLs

We used the random permutation to test the significance of the differentially expressed genes combined MI miRNA and TF to form FFLs, same method as our previous studies^19^,^20^. Detailly, we randomly selected the same number of genes from the human protein-coding genes and calculated the number of FFLs among 31 differentially expressed TFs, 83 MI miRNAs and those randomly selected genes, then calculated the number of FFLs. We repeated this 10000 times, and set the P-value as the proportion of the random results that had no less than the number of FFLs observed in the MI recurrence related network.

## Author Contributions

A.Y.G. conceived and designed the study and method. Y.L., V.L.S., H.Z. and H.H. performed the procedure and analyzed the data. V.L.S. and Y.L. wrote the manuscript. H.L. revised the manuscript and offered valuable suggestions on the script and the method. All authors reviewed the manuscript.

## Supplementary Material

Supplementary Information Supplementary Information

Supplementary Information Supplementary Information

Supplementary Information Supplementary Information

## Figures and Tables

**Table 1:** Summary of the predicted and verified MI specific miRNA and TF network

Module types	Predicted network				Verified network			
	Modules	Genes	miRNAs	TFs	Modules	Genes	miRNAs	TFs
TF-FFL	1424	63	61	88	41	12	16	15
miRNA-FFL	815	61	59	146	62	23	22	36
Composite-FFL	143	51	32	32	6	5	4	4
FBL	71	-	38	50	15	-	10	12

**Table 2:** Canonical pathway enrichment of genes in the MI regulatory network

Biological process annotation of TFs		Pathway enrichment of genes	
Biological process	P-Value	Pathway	P-Value
Cytokine mediated signaling pathway	1.0E-04	NFAT and Hypertrophy of the heart	5.6E-03
Response to hypoxia	3.2E-03	Focal adhesion	7.3E-05
Regulation of angiogenesis	4.6E-03	ECM-receptor interaction	7.2E-03
JAK-STAT cascade	7.8E-04	Pathways in cancer	6.6E-04
Anti-apoptosis	1.7E-03	Bladder cancer	6.6E-03
TGFβ signaling pathway	3.9E-3	Prostate cancer	8.8E-3
