# Supplementary material for: MiRNA and TF co-regulatory network analysis for the pathology and recurrence of myocardial infarction
Source: Sci Rep. 2015 Apr 13;5:9653. doi: 10.1038/srep09653 (PMC4394890; doi:10.1038/srep09653)
Supplement: Supplementary Information [file srep09653-s1.pdf]

Supplementary information

MiRNA and TF co-regulatory network analysis for the pathology and recurrence of myocardial infarction

Ying Lin[a], Vusumuzi Leroy Sibanda[a], Hong-Mei Zhang, Hui Hu, Hui Liu[*] and An-Yuan Guo[*]

Hubei Bioinformatics & Molecular Imaging Key Laboratory, Department of Biomedical Engineering, Key Laboratory of Molecular Biophysics of the Ministry of Education, College of Life Science and Technology, Huazhong University of Science and Technology, Wuhan, 430074, China

Supplementary Tables

Supplementary Table S1: Basic information of MI related miRNAs curated from literature and databases

MI miRNA	Location (Chr:start-end[strand])	Host gene	Taxonomy conservation
miR-186-5p	chr1: 71533314-71533399 [-]	ZRANB2	vertebrates
miR-1	chr20: 61151513-61151583 [+]	C20orf166	vertebrates
miR-29c-3p	chr1: 207975197-207975284 [-]	Intergenic	vertebrates
miR-26b-5p	chr2: 219267369-219267445 [+]	CTDSP1	vertebrates
miR-210-3p	chr11: 568089-568198 [-]	Intergenic	vertebrates
miR-21-5p	chr17: 55273409 - 55273480	Intergenic	mammals
miR-150-5p	chr19: 54695854 - 54695937	Q6ZNZ9_HUMAN (antisense)	vertebrates
miR-451a	chr17: 24212513 - 24212584	Intergenic	mammals
miR-29a-3p	chr7: 130561506-130561569 [-]	Intergenic	vertebrates
miR-98-5p	chrX: 53583184-53583302 [-]	HUWE1	vertebrates
miR-24-3p	chr9: 97848303-97848370 [+]	C9orf3	vertebrates
miR-18a-5p	chr13: 92003005-92003075 [+]	Intergenic	mammals
miR-29b-3p	chr7: 130562218-130562298 [-]	Intergenic	mammals
miR-30a-5p	chr6: 72113254-72113324 [-]	Intergenic	vertebrates
miR-31-5p	chr9: 21512114-21512184 [-]	Intergenic	vertebrates
miR-140-5p	chr16: 69966984-69967083 [+]	WWP2	vertebrates
miR-208a-3p	chr14: 23857805-23857875 [-]	MYH6	vertebrates
miR-126-3p	chr9: 139565054-139565138 [+]	EGFL7	vertebrates
miR-214-3p	chr1: 172107938-172108047 [-]	DNM3	vertebrates
miR-195-5p	chr17: 6920934-6921020 [-]	Intergenic	mammals
miR-23b-3p	chr9: 97847490-97847586 [+]	C9orf3	mammals
miR-200b-3p	chr1: 1102484-1102578 [+]	Intergenic	mammals

miR-155-5p	chr21: 26946292-26946356 [+]	Intergenic	vertebrates
miR-192-5p	chr11: 64658609-64658718 [-]	Intergenic	vertebrates
miR-380-3p	chr14: 101491354-101491414 [+]	Intergenic	vertebrates
miR-125a-5p	chr19: 52196507-52196592 [+]	Intergenic	vertebrates
miR-212-3p	chr17: 1953565-1953674 [-]	Intergenic	mammals
miR-223-3p	chrX: 65238712-65238821 [+]	Intergenic	vertebrates
miR-1231	chr1: 201777739-201777830 [+]	NAV1	vertebrates
miR-575	chr4: 83674490-83674583 [-]	SCD5	mammals
miR-933	chr2: 176032361-176032437 [-]	ATF2	vertebrates
miR-320a	chr8: 22102475-22102556 [-]	Intergenic	vertebrates
miR-122-5p	chr18: 56118306-56118390 [+]	Intergenic	mammals
miR-375	chr2: 219866367-219866430 [-]	Intergenic	vertebrates
miR-499a-5p	chr20: 33578179-33578300 [+]	MYH7B	vertebrates
miR-423-5p	chr17: 28444097-28444190 [+]	NSRP1	vertebrates
miR-494-3p	chr14: 101495971-101496051 [+]	Intergenic	mammals
miR-497-5p	chr17: 6921230-6921341 [-]	Intergenic	mammals
miR-92a-3p	chr13: 92003568-92003645 [+]	Intergenic	mammals
miR-16-5p	chr13: 50623109-50623197 [-]	DLEU2	mammals
miR-15a-5p	chr13: 50623255-50623337 [-]	DLEU2	mammals
miR-15b-5p	chr3: 160122376-160122473 [+]	SMC4	mammals
miR-132-3p	chr17: 1953202-1953302 [-]	Intergenic	vertebrates
miR-30c-5p	chr1: 41222956-41223044 [+]	NFYC	vertebrates
miR-145-5p	chr5: 148810209-148810296 [+]	Intergenic	vertebrates
let-7a-5p	chr9: 96938239-96938318 [+]	Intergenic	vertebrates
let-7b-5p	chr22: 46509566-46509648 [+]	RHO	mammals
let-7c-5p	chr21: 17912148-17912231 [+]	LINC00478	vertebrates
let-7d-5p	chr9: 96941116-96941202 [+]	PRPF31	vertebrates
let-7e-5p	chr19: 52196039-52196117 [+]	Intergenic	vertebrates
let-7f-5p	chr9: 96938629-96938715 [+]	PRPF31	vertebrates

let-7g-5p	chr3: 52302294-52302377 [-]	WDR82	mammals
let-7i-5p	chr12: 62997466-62997549 [+]	C12orf61-001 (antisense)	mammals
miR-103a-3p	chr20: 3898141-3898218 [+]	PANK2	mammals
miR-106a-5p	chrX: 133304228-133304308 [-]	Intergenic	mammals
miR-107	chr10: 91352504-91352584 [-]	PANK1	mammals
miR-128-3p	chr2: 136422967-136423048 [+]	R3HDM1	vertebrates
miR-139-5p	chr11: 72326107-72326174 [-]	PDE2A	vertebrates
miR-143-3p	chr5: 148808481-148808586 [+]	Intergenic	vertebrates
miR-149-5p	chr2: 241395418-241395506 [+]	GPC1	vertebrates
miR-151-5p	chr8: 141742663-141742752 [-]	PTK2	vertebrates
miR-152-3p	chr17: 46114527-46114613 [-]	COPZ2	vertebrates
miR-181b-5p	chr1: 198828002-198828111 [-]	PRPF31	vertebrates
miR-181a-5p	chr9: 127454721-127454830 [+]	NR6A1(antisense)	vertebrates
miR-185-3p	chr22: 20020662-20020743 [+]	TANGO2	vertebrates
miR-191-5p	chr3: 49058051-49058142 [-]	DALRD3	vertebrates
miR-193a-5p	chr17: 29887015-29887102 [+]	Intergenic	mammals
miR-197-3p	chr1: 110141515-110141589 [+]	Intergenic	mammals
miR-20a-5p	chr13: 92003319-92003389 [+]	Intergenic	mammals
miR-22-3p	chr17: 1617197-1617281 [-]	Intergenic	mammals
miR-27-3p	chr19: 13947254-13947331 [-]	Intergenic	mammals
miR-361-5p	chrX: 85158641-85158712 [-]	CHM	vertebrates
miR-378a-3p	chr5: 149112388-149112453 [+]	PPARGC1B	vertebrates
miR-422a	chr15: 64163129-64163218 [-]	Intergenic	vertebrates
miR-432-5p	chr14: 101350820-101350913 [+]	RTL1	vertebrates
miR-486-5p	chr8: 41517959-41518026 [-]	ANK1	vertebrates
miR-574-3p	chr4: 38869653-38869748 [+]	FAM114A1	vertebrates
miR-503-5p	chrX: 133680358-133680428 [-]	Intergenic	mammals
miR-572	chr4: 11370451-11370545 [+]	Intergenic	mammals
miR-638	chr19: 10829080-10829179 [+]	DNM2	vertebrates

miR-663a	chr20: 26188822-26188914 [-]	Intergenic	mammals
miR-671-5p	chr7: 150935507-150935624 [+]	CHPF2	vertebrates
miR-93-5p	chr7: 99691391-99691470 [-]	MCM7	vertebrates

Taxonomy conservation: mammals: (human, mouse, rat, dog); vertebrates: (human, mouse, rat, dog, chicken, frog, fish). Antisense: miRNA and its host gene are on opposite strand. 3’UTR: miRNA locating on the 3’UTR of its host gene. The remaining miRNAs are in the intron of their host genes.

Supplementary Table S2: Basic information of MI related genes curated from literature and databases

MI gene	PUBMED ID	MI gene	PUBMED ID	MI gene	PUBMED ID
ABCA1	12165563	F7	15539626	MTTP	15136504
ACE	1328889	FABP2	16945373	NCAM1	19853610
ACE2	23630610	FASLG	15297380	NOS3	9626827
ADAMTS1	18174457	FCAR	17008591	NPPA	12514664
ADIPOQ	15063429	FGB	8565160	OLR1	15976314
ADRB1	12851615	FGFR2	11834506	OR13G1	16175505
ADRB2	11246538	GCLC	12598062	P2RY11	17135283
AGT	21474893	GCLM	12081989	PAI1	12477941
AGTR1	7934345	GJA4	12477941	PALLD	16175505
AKT1	22135402	GNAI2	18316484	PCSK9	16554528
ALOX5AP	14770184	GNB3	11116112	PDCD4	20219857
ANXA5	12200370	GP1BA	9396417	PECAM1	22578454
APOA5	18250146	GP6	11571236	PLA2G4A	21884509
APOB	3024002	HCN2	20381460	PLA2G7	9472966
APOC3	12235176	HCN4	20381460	PLAT	8042597
APOE	7966894	HMGCR	19419716	PLAU	20380835
ARG1	17369504	HMOX1	15064108	PON1	8675673
ATP2A2	18388909	HNRNPK	17975119	PPARG	12663371
BCL2	11491653	HNRPUL1	16690874	PRKCE	14654063
BDNF	24142413	HSPA4	17934269	PSMA6	16845397
BTN2A1	18704761	HSPD1	21497776	PSRC1	21242481
CASR	17374704	ICAM1	16820586	PTEN	20097771
CCL11	15186951	IGF1	7505276	PTGIS	12040339
CCL2	23925450	IL1B	15539626	PTGS2	15138244
CCR2	12719858	IL6	12123772	PURA	18344281
CCR5	11477473	IPF1	16894468	ROS1	16175505
CD14	10385492	ITGA2	9950439	SELE	12649084
CDKN2A	17634449	ITGA5	10536667	SELP	12165563
CDKN2B	22768093	ITGB3	8598867	SLC5A3	19198609
CETP	9420339	KCNJ2	18634977	SLC6A4	12081984
CFH	16630992	KCNMB1	16293791	SMAD6	22283839
COL1A1	21907695	KIF6	18222353	SOD2	17967822
COL1A2	7639329	LDLR	21975462	SPRED1	23625462
COL3A1	7639329	LGALS2	15129282	SPRY1	21932524
COL4A1	9689593	LIF	23661360	TAS2R50	17975119
COMT	15033250	LIPC	11748100	TGFB1	21219908
CPB2	12006404	LPA	12578871	THBD	11245641
CRP	17164456	LPL	16894468	THBS1	16684956
CX3CR1	15681302	LRP8	17847002	THBS2	11723011

CXCL12	24024928	LRRFIP1	20833976	THBS4	11723011
CXCL16	15836657	LTA	12426569	THPO	11257273
CYBA	9445163	LTA4H	21274744	TLR4	15302104
CYP2J2	17126841	MCL1	17490677	TNF	12818408
CYP4A11	15388642	MED13	24751643	TNFSF4	15750594
DNASE1	16877481	MHC2TA	15821736	TPO	22770769
EFNA3	18417479	MMP1	22829703	TRPC4	21427121
ENPP1	16186408	MMP2	15711638	VAMP8	16690874
ESR1	14600184	MMP3	10880048	VEGF	15937083
F12	11843842	MMP9	10880048	VEGFA	7525061
F13A1	9459313	MTAP	19272367	VKORC1	16549638
F3	15488883	MTHFR	16894468	ZNF627	18077766

Supplementary Table S3: The differential genes between recurrent patients and no recurrence patients of MI

Gene Symbol	Expression	Gene Symbol	Expression
APC	down	NEXN	down
ASAP2	down	NSFL1C	down
ATF7IP	down	P2RY12	down
BMP6	down	PGRMC1	down
C2orf88	down	PIGB	down
CAMK1D	down	PPM1L	down
CCDC18	down	PTPN12	down
CNTNAP3	down	RAB11A	down
CXCL5	down	RAB11FIP1	down
DAB2	down	RAB27B	down
DAPP1	down	RAP1GAP2	down
DLEU2	down	RAPGEF2	down
DNAJB6	down	REPS2	down
DOCK5	down	RGS18	down
EGF	down	RHD	down
ELOVL7	down	RIT1	down
ERAP2	down	RYBP	down
FAM126B	down	SDPR	down
FAM63A	down	SIPA1L2	down
FAR2	down	SIRPB2	down
FKBP1A	down	SNN	down
FKSG49	down	SNRNP48	down
FRMD3	down	SPAG9	down
HIST1H2AC	down	SSX2IP	down
HIST1H2AG	down	STON2	down
HIST1H2BD	down	TAGLN2	down
HIST1H2BE	down	TET2	down
HIST1H2BF	down	TMEM55A	down
HIST1H2BH	down	TMEM64	down
HIST1H2BI	down	TSPAN2	down
HIST2H2BE	down	TUBB1	down
HSPC159	down	TXNDC3	down
IGF2BP3	down	UBR5	down
IKBIP	down	VEPH1	down
IPO11	down	VNN3	down
KIAA0430	down	XIAP	down
KIAA1731	down	ZNF185	down
KIAA1841	down	ACVR2B	up
KIF2A	down	ANXA6	up

KLHL21	down	BZW2	up
LAMP2	down	C1orf228	up
LOC338758	down	FBXW4	up
LOC643792	down	H1F0	up
LRRK2	down	LOC401442	up
LYPLAL1	down	LRRC18	up
MFAP3L	down	RWDD2A	up
MKNK1	down	SLFN5	up
MPZL3	down	TRBV7-8	up
N4BP2L2	down	YY2	up
NEDD9	down	ZNF831	up

Up means the increasing of genes expression in recurrent patients and down means the decreasing of genes expression in recurrent patients.

Supplementary Figure S1

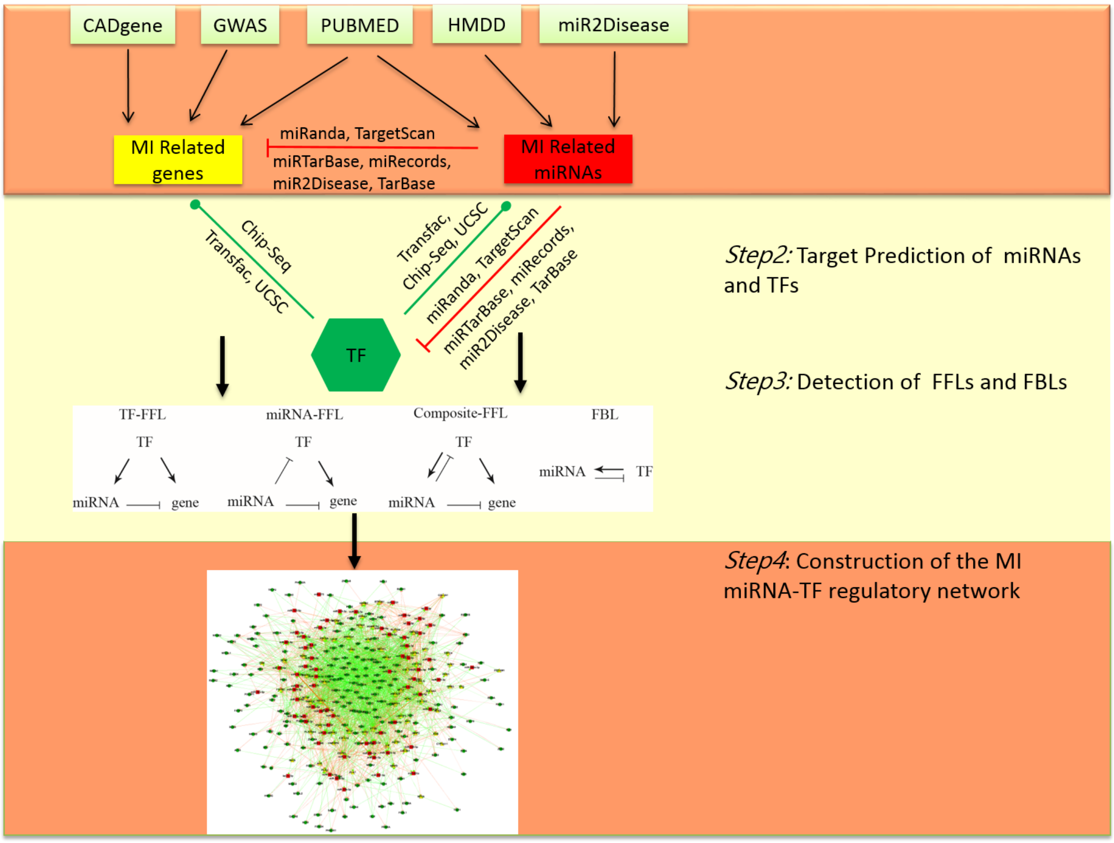

Figure S1.Workflow of the construction of the MI miRNA-TF co-regulatory network
